# Returning to the Wilderness: Potential Habitat Suitability of Non-Native Pet Birds in South Africa

**DOI:** 10.3390/biology13070483

**Published:** 2024-06-28

**Authors:** Tinyiko C. Shivambu, Ndivhuwo Shivambu, Takalani Nelufule, Moleseng C. Moshobane, Nimmi Seoraj-Pillai, Tshifhiwa C. Nangammbi

**Affiliations:** 1 Faculty of Science, Department of Nature Conservation, Tshwane University of Technology, Private Bag X680, Pretoria 0001, South Africa; shivambun@tut.ac.za (N.S.); nelufulet@tut.ac.za (T.N.); seorajpillayn@tut.ac.za (N.S.-P.); nangammbitc@tut.ac.za (T.C.N.); 2 South African National Biodiversity Institute, Pretoria National Botanical Garden, 2 Cussonia Avenue, Brummeria, Silverton 0184, South Africa; m.moshobane@sanbi.org.za

**Keywords:** biological invasions, ensemble models, establishment scenarios, invasion risk assessment, companion animals, surveillance monitoring

## Abstract

**Simple Summary:**

The global trade of pet birds has grown significantly, leading to more non-native birds being introduced into the wild. Our study examined the potential habitats and environmental factors that might allow pet birds lost and sighted in South Africa to survive outside captivity. Using geographic location data of pet birds, we predicted which areas might be suitable for various species such as the African grey parrot, Budgerigar, Cockatiel, Green-cheeked conure, Monk parakeet, and Rose-ringed parakeet. We found that a significant portion of South Africa could support these species, with some areas being highly suitable. The study highlighted how pet birds could escape or be released into the wild due to human activities, and that urban areas are particularly at risk. This research emphasizes the need for careful monitoring to prevent these birds from becoming invasive, which could impact local ecosystems and biodiversity.

**Abstract:**

The global trade of non-native pet birds has increased in recent decades, and this has accelerated the introduction of invasive birds in the wild. This study employed ensemble species distribution modelling (eSDM) to assess potential habitat suitability and environmental predictor variables influencing the potential distribution of non-native pet bird species reported lost and sighted in South Africa. We used data and information on lost and found pet birds from previous studies to establish and describe scenarios of how pet birds may transition from captivity to the wild. Our study revealed that models fitted and performed well in predicting the suitability for African grey (*Psittacus erithacus*), Budgerigar (*Melopsittacus undulatus*), Cockatiel (*Nymphicus hollandicus*), Green-cheeked conure (*Pyrrhura molinae*), Monk parakeet (*Myiopsitta monachus*), and Rose-ringed parakeet (Psittacula krameri), with the mean weighted AUC and TSS values greater than 0.765. The predicted habitat suitability differed among species, with the suitability threshold indicating that between 61% and 87% of areas were predicted as suitable. Species with greater suitability included the African grey, Cockatiel, and Rose-ringed parakeet, which demonstrated significant overlap between their habitat suitability and reported lost cases. Human footprint, bioclimatic variables, and vegetation indices largely influenced predictive habitat suitability. The pathway scenario showed the key mechanisms driving the transition of pet birds from captivity to the wild, including the role of pet owners, animal rescues, adoption practices, and environmental suitability. Our study found that urban landscapes, which are heavily populated, are at high risk of potential invasion by pet birds. Thus, implementing a thorough surveillance survey is crucial for monitoring and evaluating the establishment potential of pet species not yet reported in the wild.

## 1. Introduction

Globalization and increased human movement have accelerated the introduction of non-native species into novel and critically threatened ecosystems [1,2,3]. This has presented considerable challenges to local biota and ecological balance [4,5]. In today’s era of globalization, the commercialization of goods, including wildlife products such as animal products and live pets, contributes to billions of dollars in turnover profit [6,7,8,9]. This has led to a global increase in the trade of live animals across various taxa, including birds, mammals, reptiles, amphibians, insects, fishes, gastropods, and spiders [10,11,12,13]. This surge in trade exacerbates illicit activities involving endangered and unregulated non-CITES pet species and contributes to an increase in the introduction of invasive species [8,14,15,16]. In Europe alone, over 20 million pet reptiles were imported between 2004 and 2011 [17], and in Canada, more than 20 million wildlife were imported between 2007 and 2017 [18]. This complex chain of trading live animals is a global phenomenon linked to pet escapes and releases, leading to biological invasions worldwide [15,19,20].

Biological invasion has emerged as one of the widely discussed concepts, referring to the introduction of non-native species through various pathways into new regions, with the potential to cause environmental and socioeconomic impacts [21,22,23]. There have been several cases of non-native pet taxa reported invasive in non-native ranges, more especially those with the probability of escaping captivity [15,24,25]. For example, birds are among groups that have the potential to escape captivity and are more likely to establish invasive populations if released into the wild [25,26,27]. This is likely because the majority of bird species, particularly vagrant invasive, are not sensitive to changing environments and have displayed the phenomenon of plasticity in feeding behavior, enabling them to enhance their adaptability [28,29,30]. Examples of such species include global invasive pet birds such as Common myna (*Acridotheres tristis*), Common starling (*Sturnus vulgaris*), Monk parakeet (*Myiopsitta monachus*), Rock dove (*Columba livia*), and the Rose-ringed parakeet (*Psittacula krameri*) [28,30,31]. These bird species are among the most kept pets in the world, and many of them are designated as of least concern or non-CITES, linking them to relaxed regulation, although their impacts are of concern to native biodiversity [9,10,12,32]. For example, the establishment of a feral population of Monk parakeets has been associated with infrastructure damage, while Rose-ringed parakeets have been associated with displacement of native cavity-nesters from nests [33,34,35]. In this regard, the results on homing mechanisms and movement routes of different species of animals in an unknown habitat are significant [36,37].

Given that most pet birds either escape or are released from captivity [27], there is a lack of information regarding their distribution patterns, including the size of suitable home ranges and the environmental factors contributing to their distribution and potential invasion. The solution to this question lies in utilizing various ecological models, such as species distribution modelling (SDM) [38,39]. SDM has emerged as a pivotal tool in ecological research, encompassing risk assessment [40], climate change studies [41], and early detection of alien invasive species and pathways management [42]. It provides a quantitative framework to predict and analyze the spatial distribution of native and non-native species, relying on environmental variables [43,44]. SDM involves using statistical and computational techniques to analyze environmental variables and their relationship to the presence or absence of a particular species [45,46]. Assessing the environmental conditions where the species occurs, SDM aims to generate models that can predict suitable habitats for the species across a larger geographic scale [46,47]. The importance of utilizing SDM for invasive species lies in its ability to provide valuable insights and predictions about non-native organisms’ potential spread and impact in new environments [48,49,50]. SDM has been applied to causative vagrant invasive bird species across the nations, e.g., the Rose-ringed parakeet in Marseille, France [51], House crow in South Africa (*Corvus splendens*) [52], and Asian pied starling (*Sturnus contra*) globally [53]. The SDM modelling approach has become valuable for understanding invasive species’ ecological niche, conservation planning, and assessing the potential impacts of environmental changes on biodiversity [40,50].

South Africa is home to 971 recorded bird species, including 98 endemics, representing 9% of the global avifauna [54]. Additionally, there are 14 known alien invasive bird species in the region, although further quantification is needed (https://invasives.org.za/national-list-of-invasive-bird-species/, accessed on 10 May 2024). These bird species are kept as pets and traded for financial gain [12]. Some of these species, initially involved in the pet trade or held in captivity for companionship, end up in the wild, forming a self-sustaining population that poses a threat to native biodiversity, e.g., the Rose-ringed parakeet, Rock dove, and Common myna [55,56,57]. This includes the Monk parakeet, which is recognized as one of the most problematic invasive species established in over 16 countries [31]. There have been incidents of lost and sighted pet birds, including invasive species, reported in South Africa [58]. However, there is insufficient information regarding their potential spread and the factors that might facilitate their establishment in both invaded and previously uninvaded areas. This study employed the ensemble Species Distribution Modelling (eSDM), aiming to explore the potential distribution patterns, habitat suitability, and factors likely to facilitate the establishment of non-native lost and sighted pet birds. Furthermore, we also described pathways scenarios of lost and found pet birds, associating them with species that are likely to establish populations in the wild. Employing the eSDM technique on non-native pet birds reported lost and sighted will help uncover the ecological factors shaping the potential distribution patterns of these species.

## 2. Materials and Methods

### 2.1. Geographical Study Areas 

**Figure 1:**
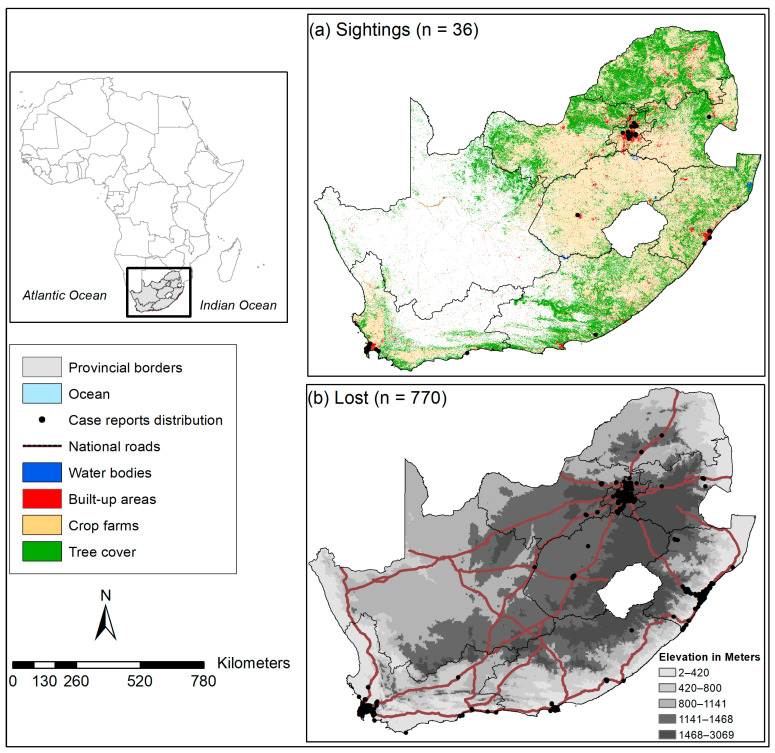
A map illustrating the geographic study area showing the land-use cover types and elevation in regions where six non-native pet birds have either been (**a**) observed outside captivity or (**b**) reported as lost in South Africa [58]. It includes national roads (N1–N4, N7–N10, N12, N14) and built-up areas as indicators of human footprint activities.

The geographical focus area of this study is South Africa, a country located in the southernmost part of Africa, encompassing the entire southern tip of the continent (Figure 1). It shares its borders with Namibia to the northwest, Botswana and Zimbabwe to the north, Mozambique, and two landlocked countries (Lesotho, and Eswatini, formerly known as Swaziland). It also shares borders with the Indian and Atlantic Oceans to the east and west, respectively ([59], Figure 1). The country features varied topography, including coastal plains, mountains, and plateaus. The Drakensberg Mountain range runs along the eastern border, with mountain peaks reaching over 3400 m above sea level and a variety of species adapted to this landscape ([59], Figure 1). The country has varied climatic conditions, ranging from Mediterranean, which occurs mostly in Western Cape Province, with mild, wet winters and warm, dry summers and heavy rainfall in winter. This is followed by the Subtropical climate, which mostly occurs in eastern parts of the country, including coastal regions of KwaZulu-Natal Province, with hot and humid summers, mild winters, and rainfall throughout the year, with a peak in summer. The northwestern parts, including the Kalahari Desert and parts of Northern Cape Province, experience a Desert and Semi-desert climate with low rainfall in summer and variation in temperatures between day (hot) and night (cold) [59]. The interior plateau climate (highveld) occurs in the central parts of the country, particularly in Johannesburg and Pretoria, with rainfall concentrated in summer and moderate temperatures, with colder winters and warmer summers. Finally, the Savannah climate is concentrated in the country’s northeastern regions, including parts of Limpopo and Mpumalanga provinces, with distinct wet and hot seasons in summer and a dry season in winter [59]. 

### 2.2. Species Selection Criteria 

**Figure 2:**
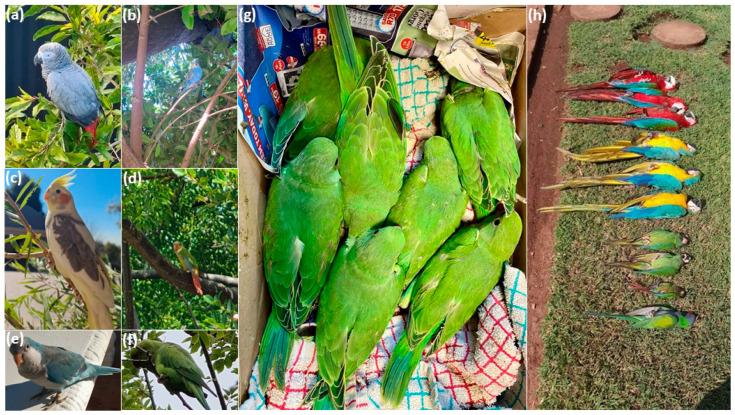
Photographs of pet bird species selected for SDM and reported lost and seen outside captivity in the field and residential areas for (**a**) African grey parrot (*Psittacus erithacus*), (**b**) Budgerigar (*Melopsittacus undulatus*), (**c**) Cockatiel (*Nymphicus hollandicus*), (**d**) Green-cheeked conure (*Pyrrhura molinae*), (**e**) Monk parakeet (*Myiopsitta monachus*), (**f**) Rose-ringed parakeet (*Psittacula krameri*), (**g**) flock of Rose-ringed parakeet found in a nest built in dead wood, and (**h**) those found dead from bee bites [top to bottom: Harlequin macaw (cross-breed), Blue-and-yellow macaw (*Ara ararauna*), Blue-winged macaw (*Primolius maracana*), Green-cheeked conure (*Pyrrhura molinae*), and Lord Derby’s parakeet (*Psittacula derbiana*)]. Photographs sourced from lost and sighted pet bird advertisements [58].

For this study, we used a dataset of non-native pet bird species reported lost, sighted, and found across South Africa between September 2011 and December 2023, covering nine provinces ([58], Figure 1). These are case reports from pet traders or owners (enthusiasts or hobbyists) who reported their pet birds lost (missing) or sighted to the dedicated lost-and-found advertisement platforms in South Africa (Figure 2), e.g., ParrotAlert (https://www.parrotalert.com/lost-and-found.php, accessed on 13 October–31 December 2023). We selected lost and sighted pet bird species from a study by Shivambu et al. [58] for SDM based on the following criteria: (1) species with a history of invasion elsewhere and introduction pathway, (2) sighted or distribution records in both or either introduced or native ranges [60], (3) potential environmental and socio-economic impacts [31,52,61], (4) a high number of lost and sighted case reports, and (5) availability in the South African pet trade [12], following selection criteria by Chucholl [62]. The availability of pet birds was evaluated based on Chucholl’s [62] selection criteria: (1) “very common”—refers to species consistently accessible across various trade sources, spanning over four provinces or involving more than three online platforms, and generally available in substantial quantities; (2) “common”—pertains to species regularly found in at least one trade source, across multiple provinces or online platforms, and in significant quantities; (3) “rare”—species occasionally available in either one source of trade, a limited number of provinces, or online platforms, and typically in low quantities; and (4) “very rare”—refers to species that are available for a brief duration in either a single trade source (online or at a pet store), in fewer than four provinces, or across less than three online platforms, typically in limited quantities. As a result, six reported lost and sighted non-native pet bird species were selected for this study: African grey, Budgerigar, Cockatiel, Green-cheeked conure, Monk parakeet, and Rose-ringed parakeet (Figure 2). Despite being reported as lost and sighted, species such as Common starling, Common myna, and Rock dove were excluded from this study because they have fewer reports and were already considered in the previous study conducted in South Africa [52]. 

### 2.3. Species Occurrence Records Download and Cleaning 

To develop the SDM, it is necessary to utilize occurrence records from either native or introduced ranges, as indicated by Steiner et al. [63]. In this study, we downloaded current global spatial occurrence records for six selected pet bird species reported lost and sighted in South Africa from the Global Biodiversity Information Facility (GBIF: https://www.gbif.org/, accessed on 17 January 2024, [64,65,66,67,68,69]), using the ‘rgbif’ package in R statistical environment [70,71] and manual download. Here, we refer to “spatial occurrence records” as georeferenced data points that indicate the locations where individuals of a particular species have been observed. The GBIF encompasses the most extensive occurrence dataset compiled from verified data from accredited sources worldwide, including international herbariums, museums (including virtual), institutions, and published literature [72,73,74]. We used GBIF records from museums, zoological collections, ornithological databases, and published scientific literature for this study, given that species identification and locations were identified and confirmed. The present occurrence records ranged between 6383 and 492,196 and were downloaded between 2010 and 2024. The quality assessment and cleaning of distribution records were conducted using ‘Biogeo’ [75] and ‘CoordinateCleaner’ [76] packages in R and using ArcGIS [77]. This process involved removing records with duplicated or overlapping occurrences and those with missing latitude or longitude coordinates within a 10 arc-minute grid cell. The outcome ensured that only one occurrence point was retained per 10 arc-minute pixels [78]. Additionally, occurrences extending beyond contiguous political boundaries were excluded using the ArcGIS, ‘Biogeo’, and ‘CoordinateCleaner’ packages in R, following the approach outlined by Robertson et al. [75]. This procedure prevents autocorrelation and spatial bias of occurrence records, substantially improving model precision and prediction reliability [46,79,80]. As a result, a subset of between 924 and 8185 occurrence records was used for modelling. Although records for species such as Budgerigar and Cockatiel were low, their inclusion in this study was important due to their successful establishment from lost captives in other studies [81].

### 2.4. Climatic Data and Environmental Variable Selections 

We employed the ‘sdm’ package [46] in the R statistical environment to produce ensemble ecological niche models for six selected pet bird species reported lost and sighted in South Africa. We downloaded a set of 19 bioclimatic variables (https://www.worldclim.org/, accessed on 13 January 2024, [82,83]) with a spatial resolution of 10 arc-min in GeoTIFF format. In addition, we downloaded water vapor pressure (kPa) from WorldClim (https://www.worldclim.org/, accessed on 14 January 2024) along with elevation variables at a 10 arc-min spatial resolution in GeoTIFF format, derived from the Shuttle Radar Topography Mission [84]. The Normalized Difference Vegetation Index (NDVI) for the year 2024, serving as a measure of biome greenness, was obtained from Copernicus Global Land Service version V2.0.1 (tile NDVI300_202401010000; [85]). The initial resolution at 300 m was transformed to a spatial resolution of 10 arc-min to align with the resolution employed for other variables in this study. We employed these environmental variables as predictors to assess the suitability of each pet bird species. These variables were selected due to their potential influence on direct physiological and ecological processes that could impact the survival and distribution of the bird species [86,87,88,89]. In addition, ambient humidity linked to water vapor pressure is crucial for nest conditions, as nest humidity is influenced by how water vapor is retained within the nest [90,91,92]. We tested correlations among 19 bioclimatic variables using the Variance Inflation Factor function in R (VIF; [93]) and Pearson (r) correlation coefficients to identify multicollinearity. This is because multicollinearity has an impact on model performance and prediction, as well as effects on SDM, such as model uncertainty and overfitting [46,94]. A threshold value of ρ = |0.7| was considered as a cut-off point to choose variables that were not highly collinear, i.e., variables with ρ < 0.7 [94]. As a result, eight non-collinear bioclimatic variables were included when building the model for each species (Table 1).

In addition to environmental predictor variables, we included the global map of the human influence/impact (human footprint) on the earth’s climate, ecosystems, wildlife, and human health and welfare on a global scale [95,96]. Human footprint variables encompass a wide range of factors, including population density, land use and land cover, infrastructure presence (e.g., roads and buildings), access to natural areas, electric power infrastructure, light pollution, and agricultural activities. Each of these factors contributes to quantifying the extent and intensity of human impact on the environment [96]. Incorporating the human footprint in this study was deemed essential, as most non-native invasive pet bird species have demonstrated a tendency to be widely distributed across the globe in connection with human activities [28,30,56,97]. The human footprint index for the year 2020 (tile HF3-2) (https://storage.googleapis.com/hii-export/2020-01-01/hii_2020-01-01.tif, accessed on 10 January 2024; [96]) in GeoTIFF format was downloaded and integrated into the model at a 10 arc-minute spatial resolution.

**Table 1:** A set of non-collinear environmental variables used as predictor variables for modelling six non-native pet bird species reported lost and sighted outside captivity.

Variables Code	Variable Description	Sources
BIO2	Mean Diurnal Range (Mean of monthly (max temp − min temp))	(WorldClim, [82,83])
BIO3	Isothermality (BIO2/BIO7) (×100)	(WorldClim, [82,83])
BIO5	Max Temperature of Warmest Month	(WorldClim, [82,83])
BIO8	Mean Temperature of Wettest Quarter	(WorldClim, [82,83])
Bio9	Mean Temperature of Driest Quarter	(WorldClim, [82,83])
Bio15	Precipitation Seasonality (Coefficient of Variation)	(WorldClim, [82,83])
Bio18	Precipitation of Warmest Quarter	(WorldClim, [82,83])
Bio19	Precipitation of Coldest Quarter	(WorldClim, [82,83])
WVP	Water Vapor Pressure (kPa)	(WorldClim, [83])
Elv	Elevation	(WorldClim, [83,84])
NDVI	Normalized Difference Vegetation Index	[85]
HF3	Human footprint	[95,96]

### 2.5. Model Fitting and Performance 

The potential distribution models for six lost and sighted pet bird species were assessed using the R statistical software [71]. SDMs were produced using three algorithms, correlating occurrence records of six pet bird species with environmental predictor variables using the ‘sdm’ package [46]. Several studies have emphasized the importance of including both presence occurrence and absence records for species in fitting distribution models [46,47,98]. Thus, in this study, 1000 pseudo-absence records were randomly drawn from a defined background of present records at average runs of 100 bootstrap replications [47,99,100]. The algorithms employed in this study included a combination of both popular machine-learning and regression-based methods capable of handling varying levels of complexity in determining the relationship between the occurrence records and the employed environmental variables. The algorithms used included the Generalized Linear Model, Random Forest, and Support Vector Machine (Table 2). Calibration for each of the three methods was conducted using the default settings embedded in the packages from R libraries associated with the ‘sdm’ package. Models were calibrated using 70% occurrence records split for training and 30% records for testing, while predictive performance was evaluated at 100 runs of bootstrap replication to give the model adequate time to converge. 

Two widely used independent and dependent-threshold statistics, including the area under the receiver operating characteristic curve (AUC) and the true skill statistic (TSS), were employed to assess the predictive performance of individual pet bird species and construct ensemble SDMs [105,106]. The AUC and TSS were specifically used to assess the level of agreement between the presence and pseudo-absence records in predicting species suitability. The AUC threshold values range from 0 to 1, with values below 0.7 considered poor, those between 0.7 and 0.9 considered good, and values greater than 0.9 deemed excellent [107,108]. The TSS threshold values are computed using a confusion matrix and range from −1 (indicating worse performance than random) to +1 (indicating excellent performance) [105]. To predict habitat suitability for six pet bird species, an ensemble model comprising three combined algorithm methods was used to improve prediction accuracy, as demonstrated in previous studies [109,110]. To construct a single consensus ensemble model for each species, we employed a weighted method to average the output of all three methods for individual species. We utilized the Maximum Training Specificity plus Sensitivity threshold to establish the presence–absence threshold [111,112]. This threshold minimizes the average error rate for both positive and negative observations and demonstrates superior performance compared to alternative thresholds in delivering precise presence predictions [112,113]. As a result, the omission rate, sensitivity, specificity, proportion of model correctness, and model calibration were assessed to determine the habitat suitability model performance’s satisfactory accuracy, reliability, and performance levels. The habitat suitability threshold was calibrated, indicating the percentage of suitable areas for each modelled species. The percentage represented as the suitability threshold indicates the predicted extent of suitable habitat for each of the six species in South Africa. 

### 2.6. Establishment Pathway Scenarios 

Based on observations and documented occurrences [58], we developed a pathways scenario describing the mechanisms through which pet birds transition from captivity to instances of loss. The inclusion of these pathway scenarios was necessary for this study as they illustrate a range of mechanisms linked to the found-lost-establishment of pet birds in the country, aiding in the selection of suitable species for modelling. The pathway scenario highlights the key factors driving the transition of pet birds from captivity to the wild, including the roles of pet owners, animal rescues, adoption practices, and environmental suitability. This association is fundamental for understanding the processes that influence the establishment of these species and ensures that our models accurately reflect real-world dynamics. To construct the stepwise pathway scenarios, we used yEd Graph Editor software version 3.23.2 [114].

## 3. Results 

### 3.1. Model Performance 

The models fitted in this study performed well in predicting the habitat suitability for six pet bird species reported lost and sighted, with a weighted mean AUC value of 0.938 (±0.033; mean ± SD) and weighted mean TSS value of 0.765 (±0.093; mean ± SD) (Table 3, Supplementary Materials Table S1). Of the six pet bird species, the model for independent threshold, AUC, performed well for all pet birds, with the model for Budgerigar being the least performing (Table 3). The model for dependent threshold (TSS) performed well for Green-cheeked conure with a mean TSS of 0.891 (±0.019; mean ± SD). For the remaining five pet bird species, the mean TSS value ranged from 0.64 to 0.788 (Table 3, Supplementary Materials Table S1). The three algorithm methods used in fitting the model performed well for the six bird species selected (Supplementary Materials Table S1). The assessment of model performance indicates satisfactory levels of accuracy, reliability, and performance, as reflected by the omission rate (0.104 ± 0.074, mean ± SD), sensitivity, specificity, proportion of model correctness (0.892 ± 0.072), and model calibration (0.805 ± 0.065) (Supplementary Materials Table S1). For all six pet bird species, GLM, RF, and SVM methods performed significantly in fitting model prediction for both independent and dependent thresholds (Supplementary Materials Table S1). 

Of the six selected pet bird species, only the Rose-ringed parakeet has known invasive populations outside captivity, and it has been reported as lost and as an escapee (Supplementary Materials Table S2). The occurrence records for Rose-ringed parakeets are largely concentrated in Gauteng, KwaZulu-Natal, and Western Cape provinces (Supplementary Materials Figure S1). The remaining five pet bird species have varied lost case reports, ranging from 20 to 314 reports (Table 3, Supplementary Materials Figures S2–S6).

**Table 3:** A summary of the ensemble model performance for three algorithm methods generated using presence records of six non-native pet bird species reported as lost or sighted in the urban and natural ecosystems of South Africa. The number of lost case reports represents instances of bird species reported outside of captivity in South Africa. These reports were extracted from Shivambu et al. [58].

Species	Lost Case Reports	Variable		Variable of Importance (%)											
		AUC	TSS	BIO2	BIO3	BIO5	BIO8	BIO9	BIO15	BIO18	BIO19	Humidity	Elv	NDVI	HF3
African grey	314	0.968	0.826	8.30	9.94	5.67	6.22	7.85	10.52	13.81	4.63	4.73	5.34	8.3	14.69
Budgerigar	26	0.889	0.64	7.52	10.15	3.30	2.21	13.33	15.42	10.85	6.62	5.40	8.33	5.3	11.57
Cockatiel	220	0.912	0.68	5.77	9.32	3.01	2.32	14.82	8.18	10.22	9.21	7.30	7.94	4.2	17.71
Green-cheeked conure	41	0.977	0.891	9.01	6.41	3.73	3.41	6.87	7.93	16.71	12.62	5.04	4.15	10.33	11.79
Monk parakeet	20	0.943	0.788	9.41	7.42	3.64	2.75	8.63	10.11	8.54	11.71	13.02	4.41	8.11	12.25
Rose-ringed parakeet	149	0.938	0.762	5.91	7.13	3.34	1.64	12.87	11.63	9.13	14.05	7.15	3.26	9.4	14.49

### 3.2. Habitat Suitability and Importance Variables

**Figure 3:**
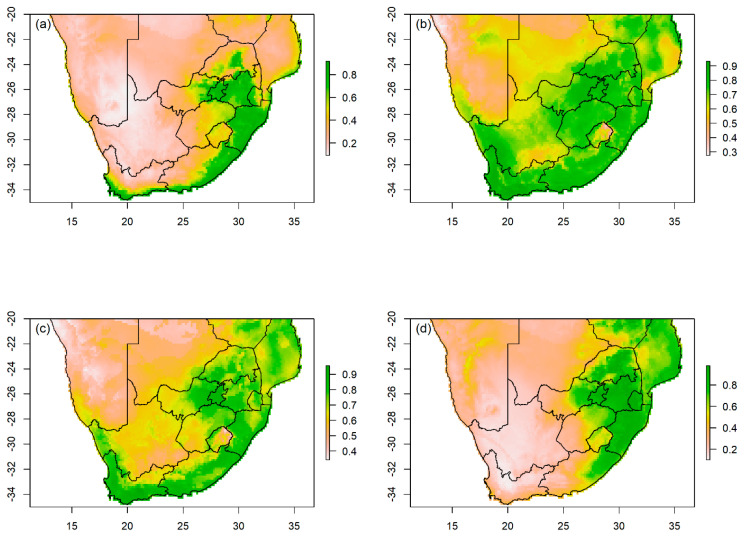
The species distribution modelling showing predicted potential suitability for (**a**) African grey parrot (*Psittacus erithacus*), (**b**) Budgerigar (*Melopsittacus undulatus*), (**c**) Cockatiel (*Nymphicus hollandicus*), and (**d**) Green-cheeked conure (*Pyrrhura molinae*) in South Africa. The suitability measure is represented by a threshold with a color ramp on the right of the map; the greener the threshold, the greater the suitability. The level of overlap between suitability and lost case report for each species is shown in Supplementary Materials Figures S2–S5.

The prediction threshold showed that 0.61 (61%) of South Africa has suitable habitat for the African grey (Supplementary Materials Table S1). The predicted suitability for this species was largely in eastern areas of South Africa, including Gauteng, Mpumalanga, KwaZulu-Natal, and Eastern Cape provinces (Figure 3a and Figure S2). This includes the coastal regions of the Western Cape Province and central and western parts of Limpopo extending towards the eastern regions of Nort West Province (Figure 3a and Figure S2). The eastern regions in the Free State Province towards the Drakensberg Mountains had suitable areas (Figure 3a and Figure S2). The reported lost cases of African grey overlapped with predicted suitability in the Gauteng, KwaZulu-Natal, Eastern Cape, and Western Cape provinces (Figure S2). The predictor environmental variables that contributed the most to the African grey model predictions included Human footprint, Bio 18, Bio 15, Bio 3, Bio 2, and NDVI (Table 3).

The prediction suitability threshold for Budgerigar showed that 0.858 (86%) of areas in South Africa are likely suitable for this species (Supplementary Materials Table S1). The model predictions indicate that nearly all regions of the country are predicted to be suitable for the species, including areas where the species was largely reported lost, particularly in Gauteng and Western Cape provinces (Figure 3b and Figure S3). The predictor variables best describing the Budgerigar suitability included Bio 15, Bio 9, Human footprint, Bio 18, Bio 3, and elevation (Table 3). 

The predicted threshold suitability showed that 0.723 (72%) of areas are likely suitable for the Cockatiel in South Africa (Supplementary Materials Table S1). All nine provinces in the country were predicted to be suitable, with less predicted suitability in Northern Cape arid regions extending towards the border with Eastern Cape and Western Cape provinces (Figure 3c and Figure S4). The reported lost case report for Cockatiel overlapped with the predicted suitability in Gauteng and Western Cape provinces (Figure S4). The predicted suitability for this species was best described by human footprint, Bio 9, Bio 18, Bio 3, Bio 19, and elevation (Table 3).

Our study showed that 0.673 (67%) of habitat is likely suitable for Green-cheeked conure (Supplementary Materials Table S1). The model predictions showed that only areas in the eastern regions of the country are suitable for this species, particularly areas in Limpopo, Gauteng, Mpumalanga, KwaZulu-Natal, and eastern coastal regions of Eastern Cape Province (Figure 3c). The predicted suitable areas overlapped with documented case reports in Gauteng and KwaZulu-Natal provinces (Figure S5). Bio 18, Bio 19, human footprint, NDVI, and Bio 2 were the most contributing environmental predictor variables in predicting the suitability for Green-cheeked conure (Table 3).

**Figure 4:**
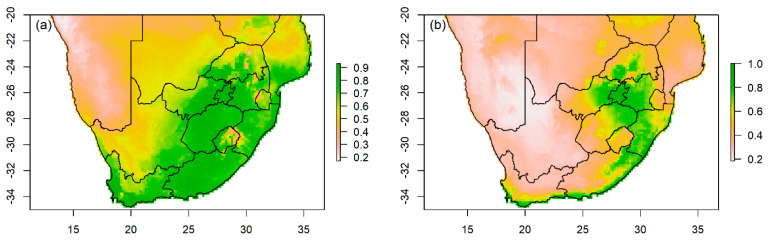
The species distribution modelling showing predicted potential suitability for (**a**) Monk parakeet (*Myiopsitta monachus*) and (**b**) Rose-ringed parakeet (*Psittacula krameri*) in South Africa. The suitability measure is represented by a threshold with a color ramp on the right of the map; the greener the threshold, the greater the suitability. The level of overlap between suitability and lost case report for each species is shown in Supplementary Materials Figures S1 and S6.

The threshold suitability showed that 0.866 (87%) of areas are likely to be suitable for the Monk parakeet in South Africa (Supplementary Materials Table S1). Almost all provinces in the country were predicted to be suitable, with seven provinces having greater suitability (Figure 4a). Provinces such as the Northern Cape and Limpopo had reduced suitability in western and northern regions, respectively (Figure 4a). There was an overlap between reported lost case reports and the predicted suitable areas, particularly in Gauteng and Western Cape provinces (Figure S6). The environmental predictor variables that best describe the predicted suitability for the Monk parakeet included humidity, human footprint, Bio 19, Bio 15, Bio 2, and Bio 9 (Table 3).

The predicted suitability threshold for Rose-ringed parakeets showed that 0.717 (72%) of predicted areas are likely suitable for this species in South Africa (Supplementary Materials Table S1). The predicted suitability for this species is largely in the eastern coastal regions of South Africa, particularly in the Eastern Cape and Western Cape provinces (Figure 4b). The suitability for this species extends to inland regions of the Mpumalanga, Free State, Gauteng, North West, and Limpopo provinces (Figure 4b). Our study showed an overlap between predicted suitable areas and lost case distribution, particularly in the concentrated regions of Gauteng, KwaZulu-Natal, and Western Cape provinces (Figure S6). The predictor variables, such as human footprint, Bio 19, Bio 9, Bio 15, NDVI, and Bio 18, contributed the most in predicting suitability for the Rose-ringed parakeet in South Africa (Table 3).

### 3.3. Pathway Scenario of Lost Pet Bird Leading to Potential Establishment

**Figure 5:**
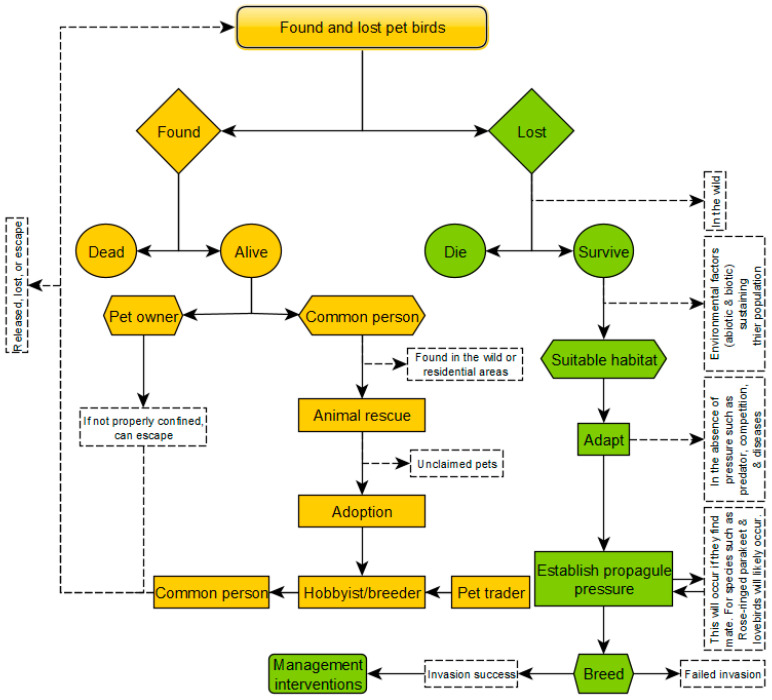
A stepwise pathways scenario describing the mechanisms by which pet birds transition from captivity to instances of found or lost, based on observations and documented occurrences in Shivambu et al. [58] and Figure 1. The yEd-constructed figure also illustrates the sequential process by which lost pet birds transition into potential invasive populations.

This study describes two-way pathways (found and lost) transition mechanisms leading to the potential establishment of some pet bird species (Figure 5). The stepwise mechanisms are described below:

*Found pet birds*: Pet birds, whether deceased (dead) or alive, can be found in various locations, including private captivity, pet stores, animal rescues, and zoos (Figure 2a–h). Often, pet birds are found alive either by their pet owners or individuals not affiliated with the ownership (hereafter referred to as common person) within the fields or residential areas (i.e., sports fields and parks).

*Response of pet owners*: If a pet bird is found alive by its owner, it is typically kept in secure confinement to prevent further escape. However, if the confinement is relaxed, the bird may escape again. In some cases, owners may release unwanted pet birds into the wild, contributing to the pool of stray or feral individuals, where some of them may be found and taken to animal rescues.

*Response of common person*: Lost birds found by individuals other than the owners in the wild or within residential areas (i.e., including sports fields and parks) may be taken to animal rescues, especially if they are identified as micro-chipped or tagged pet birds. 

*Role of animal rescues:* Animal rescues face challenges in housing a large number of pet birds, requiring manpower, medical attention, and specific dietary needs. While some birds may be successfully reunited with their owners, others may remain unclaimed and may be donated to alleviate this burden.

*Adoption and release*: Unclaimed pet birds in rescue facilities may be adopted or donated by breeders, pet traders, hobbyists, or common persons. However, this process may inadvertently lead to accidental escapes and intentional releases, particularly by inexperienced individuals. This often occurs when the adopted bird is no longer wanted, the adopter relocates, or the bird is intentionally released (i.e., to form part of native wildlife).

*Lost species*: Lost species may either die due to harsh environmental conditions or survive in the wild, often within residential areas or urban green spaces (Figure 2h).

*Survival*: Those lost species that survive typically do so in environments where favorable abiotic (e.g., suitable climate, water availability, and appropriate ambient temperature for breeding) and biotic (e.g., food sources, nesting sites, and potential breeding partners) factors are present (Figure 5).

*Adaptation*: In the absence of external pressures such as predators, competitors, and diseases, most lost species are likely to adapt due to favorable environmental conditions at the point of loss or release.

*Establishment of propagule pressure*: Species already breeding in the wild, such as Rose-ringed parakeets and lovebirds, are likely to find breeding partners, integrate with the feral populations, and expand their populations in the wild, leading to high propagule pressure (Figure 2g).

*Breeding potential*: Pet birds capable of breeding and successfully producing propagule may either become invasive or fail to do so due to unsuccessful invasion debt.

*Management interventions*: Pet birds with successful establishment are likely to spread and occupy uninvaded territories, leading to management intervention to minimize their spread and prevent future invasions. 

## 4. Discussion

### 4.1. Model Performance and Predictive Power 

The model prediction in this study provides an important insight into the distribution, habitat suitability, and potential establishment of non-native pet birds reported lost and sighted in South Africa. Through the application of ensemble SDM, the model accurately predicted the habitat suitability for six non-native pet bird species, namely the African grey, Budgerigar, Cockatiel, Green-cheeked conure, Monk parakeet, and Rose-ringed parakeet. Our results demonstrated robust model performance, with satisfactory levels of accuracy, reliability, and performance across different algorithm methods. The model for each species exhibited high predictive power, as indicated by the AUC and TSS values. Evidently, the application of eSDM combined with varied environmental variables and algorithm methods has been reported to enhance model performance and predictive reliability in many studies [109,115,116,117]. The application of SDM has become a common predictive tool for several taxa, including alien invasive pet birds [51] and those that have gone wild from the cages and become invasive [118]. Thus, the utilization of these predictive techniques in this study proved to be appropriate for non-native pet birds reported lost and sighted, incorporating a variety of applied methodologies, robust techniques, and predictor variables in alignment with the recommendations of Araújo and New [109] is necessary. As a result, our study highlighted the efficacy of eSDM in capturing the complex relationship between the environmental variables used and the distribution patterns of six selected pet bird species.

### 4.2. Environmental Variables Shaping Species Suitability

The predicted habitat suitability maps revealed distinct distribution patterns for each species, influenced by a combination of environmental factors such as bioclimatic variables, human footprint, vegetation indices, and elevation. Our study revealed that the human footprint contributed the most in predicting suitability for the African grey, Cockatiel, Monk parakeet, and Rose-ringed parakeet. These results are not surprising given that these pet bird species are commensal and depend on human environments [28,56,58,81,119]. Therefore, their overrepresentation in urban areas may be attributed to their tendency to be released there or their inability to find suitable habitats in non-native ranges. Most pet species assessed in this study are non-migratory, exhibit localized tendencies, and rely on aided movement. For example, the invasive Monk parakeet in Barcelona and Spain has a mean home range of 12.4 ± 1.22 ha and long-distance dispersions of 10 km [120,121]. Similarly, the observed travel distance of Rose-ringed parakeets in Brussels was up to 9 km between roosting and nesting sites [122]. In addition, Budgerigar, a newly emerging pet bird species, is locally establishing its population in Japan’s urban landscapes [81]. In Texas, USA, the Monk parakeet, an invasive localized to the area, shows a preference for using structures such as electricity utility for breeding [123]. A study by Banha et al. [97] applied a range of environmental variables, including the human footprint, which contributed highly to the distribution of invasive pet commensal Red-eared sliders (*Trachemys scripta elegans*) in the Iberian Peninsula. This suggests that the human footprint is highly probable to be the primary factor in predicting the distribution of various localized non-native pet species associated with human presence, thus serving as a valuable component for incorporation into habitat suitability models for alien invasive species [97,124]. 

Other predictor variables found to contribute to the habitat suitability model of the six bird species were Bio 9, Bio 18, and Bio 15. This is likely because these variables have a potential influence on direct physiological and ecological processes that could impact the survival and distribution of these bird species [86,87,88,89]. For example, Bio 9 can directly affect the availability of resources such as water and food, influencing habitat suitability for these bird species. Bio 18 can indicate the availability of water sources crucial for these bird species during periods of high temperature and potential drought stress. Lastly, Bio 15 influences the predictability and consistency of precipitation patterns, as it affects resource availability and habitat suitability for birds’ survival and breeding [125,126,127]. In addition to this, a study conducted in Puerto Rico used bioclimatic variables to predict the suitability of habitat for 10 lost and sighted pet birds, including the invasive Monk parakeet [118]. The study identified possible range expansion for these birds in both human-dominated areas and natural environments [118]. These concurrent trends and the application of similar models suggest the importance of applying such variables in studies such as this to facilitate a better understanding of potential range expansions. 

Our study found that vegetation indices contributed largely to the habitat suitability models for the Green-cheeked conure, Monk parakeet, and Rose-ringed parakeet. This could be explained by the tendency of these species to inhabit areas characterized by specific vegetation types offering a variety of food options (fruits, seeds, and foliage) important for their daily dietary needs. Additionally, these vegetation types create microhabitats suitable for nesting and roosting activities, resulting in increased species richness [125,128,129,130]. A case in point is the invasive Rose-ringed parakeets in Gauteng and KwaZulu-Natal regions, where they have invaded habitats characterized by tall, dense trees like Natal fig (*Ficus natalensis*), Natal mahogany (*Trichilia emetica*), Rose gum (*Eucalyptus grandis*), and White milkwood (*Sideroxylon inerme*), providing them with food sources, secondary nest cavities, and roosting sites [56,57]. It is, therefore, imperative to note that vegetation indices are valuable predictors for the distribution of these lost pet birds in South Africa as they provide an insight into habitat suitability, food availability, nesting sites, microclimate conditions, and water availability, all of which are important factors influencing the distribution of these bird species. 

Our study also showed that elevation may contribute to the potential distribution of lost pet birds in South Africa. Although elevation did not have much influence on the distribution of modelled species, it is known to have an influence on the environmental factors that affect habitat suitability for bird distributions [131,132]. It influences factors such as climate variation, habitat diversity, availability of nesting and roosting sites, dispersal patterns, and protection from predators [132,133]. Several studies indicated that most invasive taxa predominantly occur in subalpine and lower altitudes, e.g., the Feral cat (*Felis catus*), Common myna, Common Starling, Monk parakeet, Rock dove, and Rose-ringed parakeet [28,30,31,52,132,133]. In addition, the population of Rose-ringed parakeets in KwaZulu-Natal Province roost in elevated areas such as Umhlanga Rocks and Cowey’s Park, while during the day, they breed and forage in lower elevated areas [56]. This behavior is probably influenced by the presence of raptor species such as the Black sparrowhawk (*Accipiter melanoleucus*) and Yellow-billed kite (*Milvus aegyptius*), prompting parakeets to select roost sites in elevated locations to avoid potential predation [56]. In this study, the distribution of several lost pet bird species was least influenced by elevation, although it contributed more than some of the bioclimatic variables. This highlights the importance of including elevation as an environmental variable in the habitat suitability model to describe its potential influence on the distribution patterns of modelled non-native pet bird species.

On the other hand, our study included humidity in the model, but its contribution was low across three species and moderate across three species, namely the Rose-ringed parakeet, Cockatiel, and Monk parakeet. The inclusion of this variable was influenced by the fact that humidity plays a significant role in bird breeding and nest conditions due to its physiological influences on various aspects of their reproductive biology and habitat suitability [91,92,134]. Humidity at optimal levels is crucial for the development of bird eggs, improving incubation conditions, facilitating feather maintenance, and supporting the bird’s microclimate [135]. Thus, the potential distribution of these non-native pet bird species is likely to be influenced by the availability of optimal humidity needed during the breeding season, particularly for those species already establishing and invasive elsewhere and in South Africa, e.g., Budgerigars, Rose-ringed parakeets, and Monk parakeets [35,56,81,130]. Our study emphasizes the continued use of humidity in habitat suitability modelling, given its critical physiological role in the reproductive success and survival of bird species. 

### 4.3. Potential Invasion Hotspots and Risk Areas

The output maps generated by the habitat suitability model identified potential hotspots of invasions and high-risk areas for the establishment of non-native pet bird species in South Africa. Through the integration of eSDM and analysis of lost and sighted case reports [58], our study highlighted regions where environmental conditions are favorable for the establishment of non-native populations of pet birds. We found that all six modelled pet bird species have exhibited predicted habitat suitability in the country, with threshold suitability ranging from 61% to 87%. This suggests that large regions projected to be suitable habitats for any of these lost and sighted pet bird species in the country present a potential establishment threat.

Species with extensive suitability coverage greater than 70% included the Monk parakeet, Budgerigar, Cockatiel, and Rose-ringed parakeet. On the other hand, the Green-cheeked conure and African grey had suitability thresholds below 70%. Species of great concern are those exhibiting greater overlap between their habitat suitability maps and a large number of reported cases, such as the African grey, Cockatiel, and Rose-ringed parakeet. The predicted suitability for these species highlights regions with a high likelihood of establishment. The suitable areas identified as invasion hotspots are characterized by a combination of factors such as favorable climatic conditions, the presence of suitable habitats, and proximity to urban centers where pet bird trade and ownership are prevalent [12,58]. The previous study conducted in South Africa on pet bird trade indicated 147 species, with Budgerigar, African grey, Cockatiel, Monk parakeet, and Rose-ringed parakeet among the top 20 most traded species [12]. Of these, the latter has already been declared an invasive species in the country [136], displacing native birds from their nests, and its feral population is established in urban cities where the majority of pet shops are located [56,57]. Therefore, the predicted habitat suitability for this species is likely to facilitate its expansion beyond known established ranges, given its recent lost and sighted case reports [58]. In addition, this species is likely to thrive given that predators tend to ignore it in areas where it is established. For instance, our observations indicated that predators such as the Black sparrowhawk and Yellow-billed kite primarily prey on Rock doves rather than Rose-ringed parakeets in Durban, KwaZulu-Natal Province [56]. Conversely, the Monk parakeet, although not currently invasive, is likely to thrive in the wild, considering it is reported lost in the wild and has large habitat suitability in the country despite its invasion status elsewhere. This raises concerns, given that the species is known for its ability to outcompete native species for food and nesting sites, cause damage to infrastructure, predate crops, and create noise disturbance in urban areas [119,123,130,137]. 

Despite a large number of lost reports and favorable habitat suitability for the Cockatiel and African grey, there have been no invasion reports from locations inside and outside South Africa. It is worth noting that the Cockatiel is known to be a reservoir of zoonotic pathogens, some of which can spill over to other birds and pet owners [138]. If the majority of lost Cockatiels and African greys are not found by their owners and instead remain in the wild, there is a likelihood that they may find breeding mates, adapt, and potentially establish populations in areas where suitable habitats are available. A recent study by Nishida and Kitamura [81] indicated that the lost pet Budgerigar has transitioned into an invasive species and is now establishing itself in urban areas across Japan. Interestingly, this same species, which is among the commonly lost pet species, was also predicted to have a suitable habitat in South Africa, with an 85% suitability threshold. Although the Budgerigar is among the most kept birds worldwide, there have been many escape cases, resulting in a few self-sustaining populations in the wild [139,140,141]. Although this warrants further ground truthing to assess whether the species is established in the wild, it is important to acknowledge the potential for this species to establish a self-sustaining population in the wild, given that the predicted habitat suitability and environmental variables are associated with its preferences. Given that modelling showed suitability for all six pet bird species, it is important to note that lost pet birds, such as the African grey and Cockatiel, are less likely to survive in the wild. This is primarily because they originate from captive breeding, with most being descendants of captive individuals from several generations, which may affect their ability to adapt and survive in natural environments.

### 4.4. Lost Birds Pathways Scenarios to Species Establishment

Our study showed a comprehensive scenario analysis which reveals the pathways by which lost and sighted pet birds are likely to transition from captivity to potential establishment in the wild. This information is crucial for predicting and managing potential invasive populations. We described the key mechanisms driving this transition, including pet owners’ role, animal rescues, adoption practices, and environmental suitability. Our pathway scenario involves initial encounters with pet owners or rescue facilities, followed by potential release into the environment, adaptation to new conditions, and the eventual establishment of self-sustaining populations. This tendency is not new for pet birds; several studies have touched on some of these mechanisms, although their approach was based on specific factors of pet trade pathways that influence the release events of amphibians, birds, and reptiles [81,142,143]. Conversely, our study exclusively used proposed establishment pathway scenarios based on the habitat suitability model output for adaptation and actual reported events of pet birds reported lost and sighted in South Africa [58], with the aforementioned key mechanisms guiding the establishment process. While quantification of adaptation remains pending for other lost and sighted pet birds, species such as the Rose-ringed parakeet, which is already invasive in the country, and those established elsewhere like the Budgerigar, Cockatiel, and Monk parakeet in various regions [56,81,118,119], are considered worrisome. The adaptation and establishment scenarios of lost pet birds may be possible given that the habitat suitability model suggested that several environmental predictor variables influenced most of the modelled species’ distribution. This may lead to the emergence of problematic pet bird species with an invasion debt [144], resulting in the establishment of new populations, new invasions, and more areas invaded with greater impacts.

Consequently, lost pet bird species exhibiting predicted habitat suitability, fulfilling establishment pathway scenarios, and demonstrating the ability to breed in the wild require adaptive management interventions. Management interventions included developing targeted strategies to prevent further spread and minimize ecological impacts on native biodiversity through invasive species detection, assessment, and eradication planning [22,145,146]. Although much effort is placed into invasive plant management [147], escapees or lost pet animal taxa need to be incorporated into the management intervention and included in the NEM:BA alien and invasive species regulations list [148], or at least be included in national watchlist for non-native species before they become invasives (see [146]). This may assist in identifying four invasion indicators—introduction debt, establishment debt, spread debt, and impact debt [149]—which can be used to forecast and identify the most likely lost and sighted problematic non-native pet birds. Through tracing the pathway processes of establishment scenarios from initial loss to potential establishment, our study offers insights into the dynamics of invasion processes and highlights the importance of addressing multiple entry points to prevent further spread.

## 5. Conclusions

Our study demonstrated that all six selected pet bird species reported lost and sighted outside captivity have potentially suitable habitats in South Africa. The ensemble model performed well, predicting a large area of habitat suitability (61% to 87% threshold) overlapping with lost and sighted cases. Species showing the highest suitability overlap with lost and sighted reports may pose an invasion threat. Of most concern is the invasive Rose-ringed parakeet, which showed significant overlap between its habitat suitability and the lost and sighted reports, with its populations dominating in three provinces and persisting in the wild [58]. The predictor variables Bio 18, Bio 19, Bio 9, and Bio 3 contributed significantly to our model predictions, with the human footprint being the largest factor due to the majority of these species being commensal and urban invaders with humans’ assistance [56,118]. Many of the lost pet birds with larger habitat suitability, such as Budgerigars and Monk parakeets, have been reported to establish self-sustaining populations in the wild elsewhere [81,118,141]. As lost or sighted pet birds primarily originate from captive breeding, with most being descendants of captive individuals from several generations, this lineage may affect their ability to survive in the wild. Therefore, we recommend thorough surveillance to monitor and evaluate the establishment potential of the five remaining non-native pet birds (excluding the Rose-ringed parakeet) reported as lost and sighted but lacking documented invasion history in the country. Our study describes potential pathways through which lost and sighted pet birds transition from captivity to the wild, demonstrating various establishment scenarios which can help facilitate alien invasive management interventions. Therefore, these species should be prioritized for inclusion in the watchlist, to be potentially incorporated into the existing NEM:BA A&IS regulations list. This approach will likely promote managed and responsible pet ownership practices in the country.

## Figures and Tables

**Table 2:** A set of three algorithms employed as a statistical method for modelling six selected non-native pet bird species that went missing or were observed outside captivity.

Method Full Names	Code	Dependent Packages	References
Generalized Linear Model	GLM	STATS	[71,101]
Random Forest	RF	randomForest	[102,103]
Support Vector Machine	SVM	e1071	[104]

## Data Availability

All data used for this study are included in the Supplementary Materials.

## Supplementary Materials

The following supporting information can be downloaded at: https://www.mdpi.com/article/10.3390/biology13070483/s1, Figure S1. Ensemble species distribution modelling showing the potential distribution of the Rose-ringed parakeet (*Psittacula krameri*) in South Africa. Black dots resemble the distribution localities of Rose-ringed parakeets reported lost in the country; Figure S2. Ensemble species distribution modelling showing the potential distribution of the African grey parrot (*Psittacus erithacus*) in South Africa. Black dots resemble the distribution localities of African grey reported lost in the country; Figure S3. Ensemble species distribution modelling showing the potential distribution of the Budgerigar (*Melopsittacus undulatus*) in South Africa. Black dots resemble the distribution localities of Budgerigar reported lost in the country; Figure S4. Ensemble species distribution modelling showing the potential distribution of the Cockatiel (*Nymphicus hollandicus*) within South Africa. Black dots resemble the distribution localities of Cockatiels reported lost in the country; Figure S5. Ensemble species distribution modelling showing the potential distribution of the Green-cheeked conure (*Pyrrhura molinae*) in South Africa. Black dots resemble the distribution localities of Green-cheeked conure reported lost in the country; Figure S6. Ensemble species distribution modelling showing the potential distribution of the Monk parakeet (*Myiopsitta monachus*) in South Africa. Black dots resemble the distribution localities of Monk parakeets reported lost in the country; Table S1. A model performance for three ensembled algorithm methods generated using presence records of eight non-native pet bird species reported as having escaped from captivity in South Africa; Table S2: Geographic distribution of lost and found cases for six selected pet bird species in South Africa.
