# Supplementary material for: Returning to the Wilderness: Potential Habitat Suitability of Non-Native Pet Birds in South Africa
Source: Biology (Basel). 2024 Jun 28;13(7):483. doi: 10.3390/biology13070483 (PMC11274018; doi:10.3390/biology13070483)
Supplement: Supplementary file 1 [file biology-13-00483-s001.zip › biology-3034127-Supplementary Material.pdf]

Supplementary Material

Table S1. A model performance for three ensembled algorithm methods generated using presence records of eight non-native pet bird species reported as having escaped from captivity in South Africa.

Species	Algorithms	Threshold	AUC	Omission rate	Sensitivity	Specificity	Proportion correctness	TSS (Kappa)	Calibration
African grey	GLM	0.579	0.922	0.161	0.840	0.838	0.839	0.656	0.742
	RF	0.535	0.997	0.024	0.976	0.976	0.976	0.951	0.871
	SVM	0.765	0.972	0.061	0.939	0.939	0.939	0.878	0.796
	Ensemble	0.611	0.968	0.084	0.917	0.916	0.916	0.826	0.808
Budgerigar	GLM	0.819	0.849	0.253	0.747	0.747	0.747	0.491	0.765
	RF	0.869	0.942	0.017	0.882	0.882	0.882	0.765	0.875
	SVM	0.887	0.921	0.139	0.861	0.861	0.861	0.722	0.782
	Ensemble	0.858	0.889	0.179	0.821	0.821	0.821	0.641	0.799
Cockatiel	GLM	0.783	0.860	0.216	0.783	0.785	0.784	0.57	0.735
	RF	0.779	0.949	0.113	0.887	0.887	0.887	0.774	0.904
	SVM	0.723	0.954	0.104	0.896	0.896	0.896	0.792	0.830
	Ensemble	0.639	0.912	0.159	0.841	0.839	0.840	0.68	0.79
Green-cheeked conure	GLM	0.779	0.953	0.069	0.931	0.932	0.931	0.855	0.694
	RF	0.475	0.998	0.018	0.982	0.982	0.982	0.964	0.889
	SVM	0.545	0.991	0.035	0.967	0.963	0.965	0.929	0.848
	Ensemble	0.673	0.977	0.053	0.947	0.947	0.947	0.891	0.778
Monk parakeet	GLM	0.875	0.849	0.222	0.778	0.778	0.778	0.499	0.711
	RF	0.908	0.997	0.024	0.976	0.976	0.976	0.953	0.885
	SVM	0.816	0.982	0.059	0.941	0.941	0.941	0.882	0.812
	Ensemble	0.866	0.943	0.096	0.904	0.903	0.904	0.788	0.789
Rose-ringed parakeet	GLM	0.813	0.801	0.232	0.768	0.768	0.768	0.418	0.674
	RF	0.539	0.997	0.029	0.971	0.971	0.971	0.942	0.916
	SVM	0.706	0.982	0.052	0.948	0.948	0.948	0.896	0.822
	Ensemble	0.718	0.938	0.099	0.900	0.901	0.901	0.762	0.800

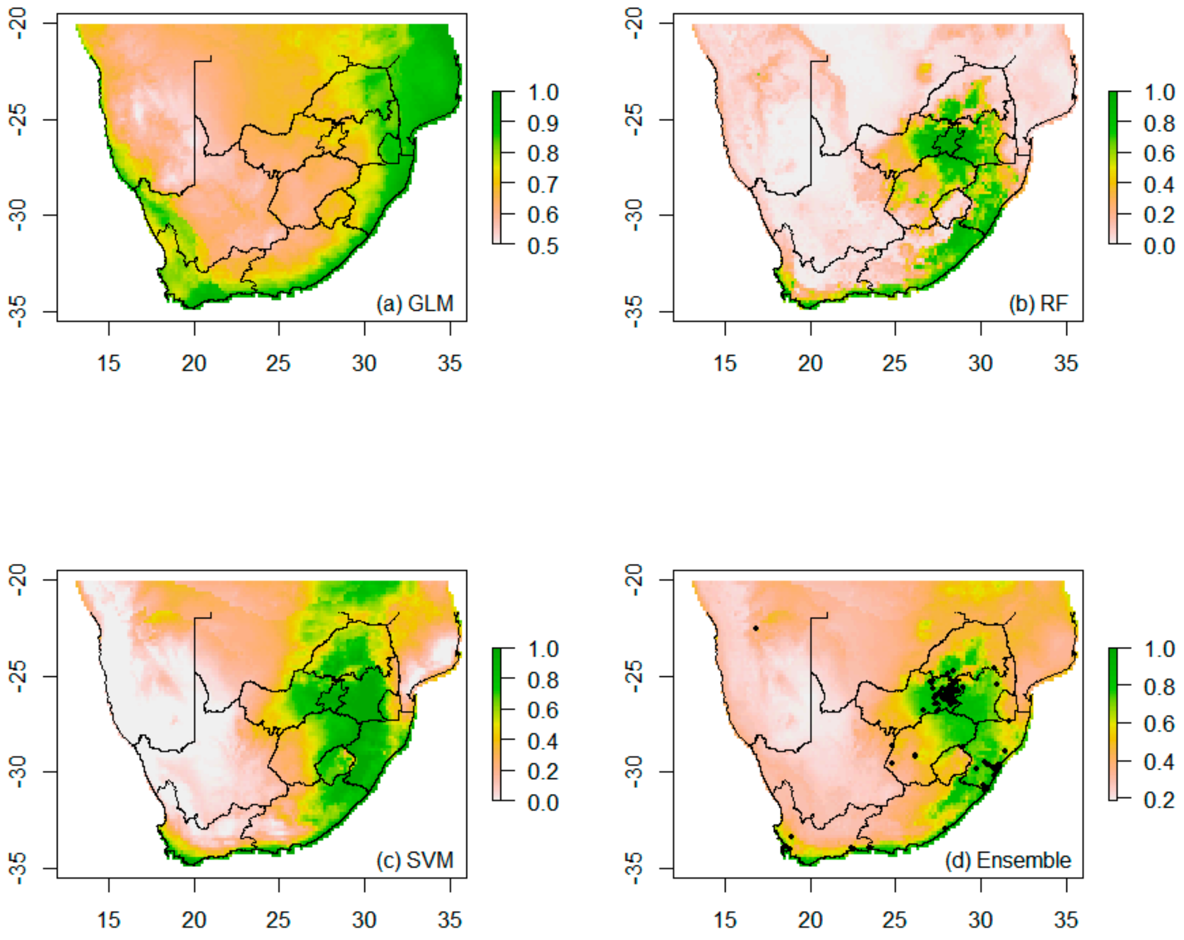

Figure S1. Ensemble species distribution modelling showing the potential distribution of the rose-ringed parakeet (*Psittacula krameri*) in South Africa. Black dots resemble the distribution localities of rose-ringed parakeets reported lost in the country.

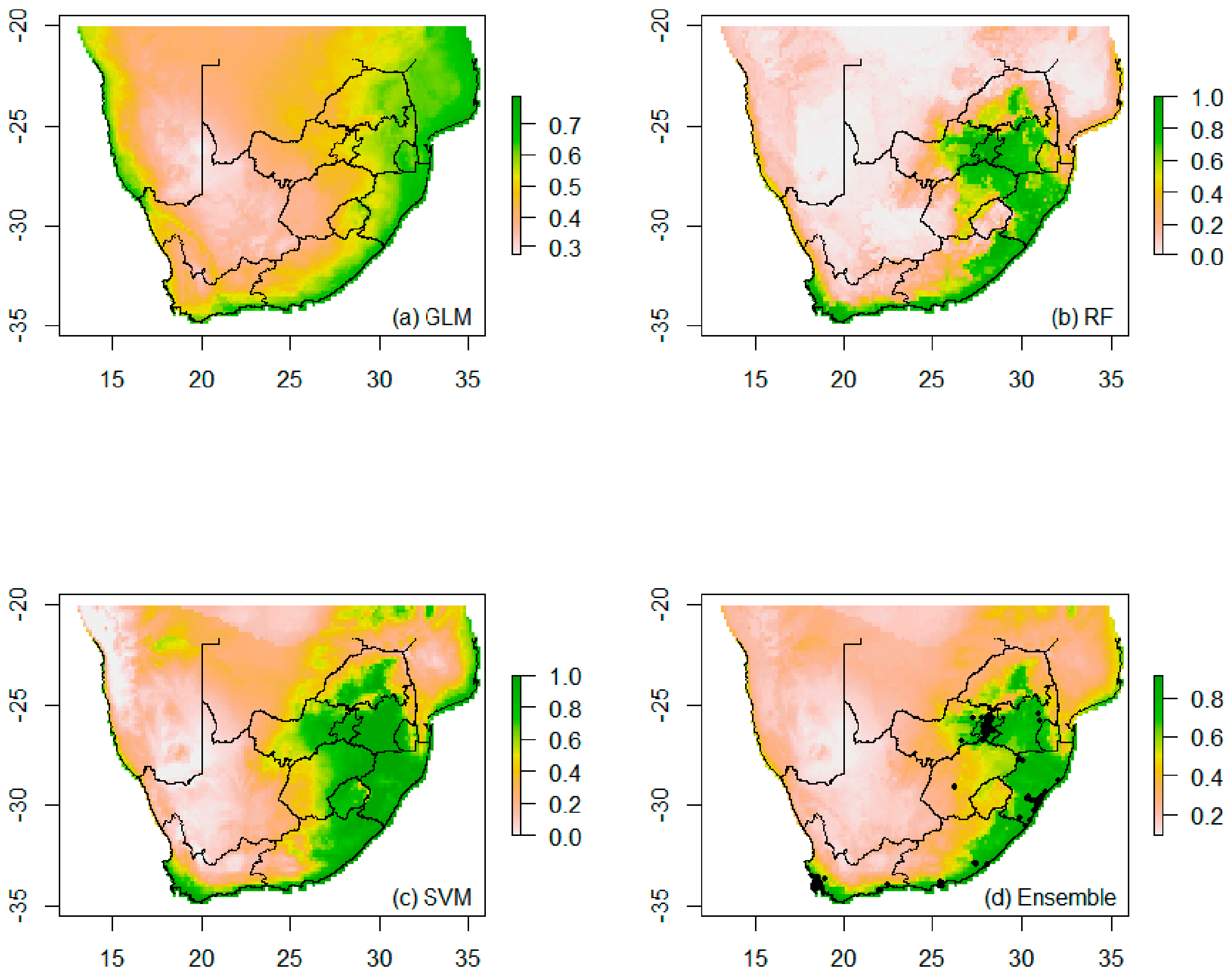

Figure S2. Ensemble species distribution modelling showing the potential distribution of the African grey parrot (*Psittacus erithacus*) in South Africa. Black dots resemble the distribution localities of African grey reported lost in the country.

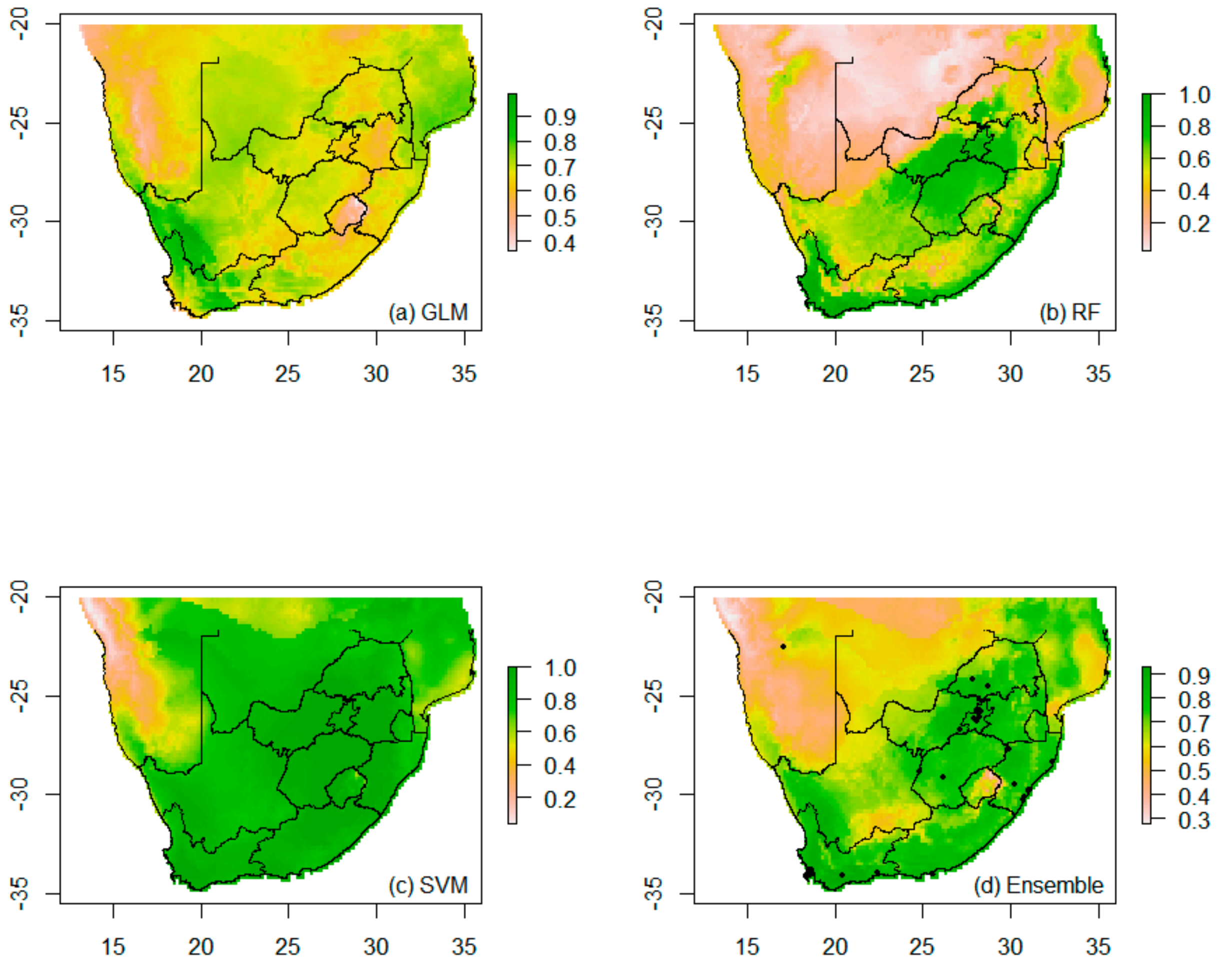

Figure S3. Ensemble species distribution modelling showing the potential distribution of the budgerigar (*Melopsittacus undulatus*) in South Africa. Black dots resemble the distribution localities of budgerigar reported lost in the country.

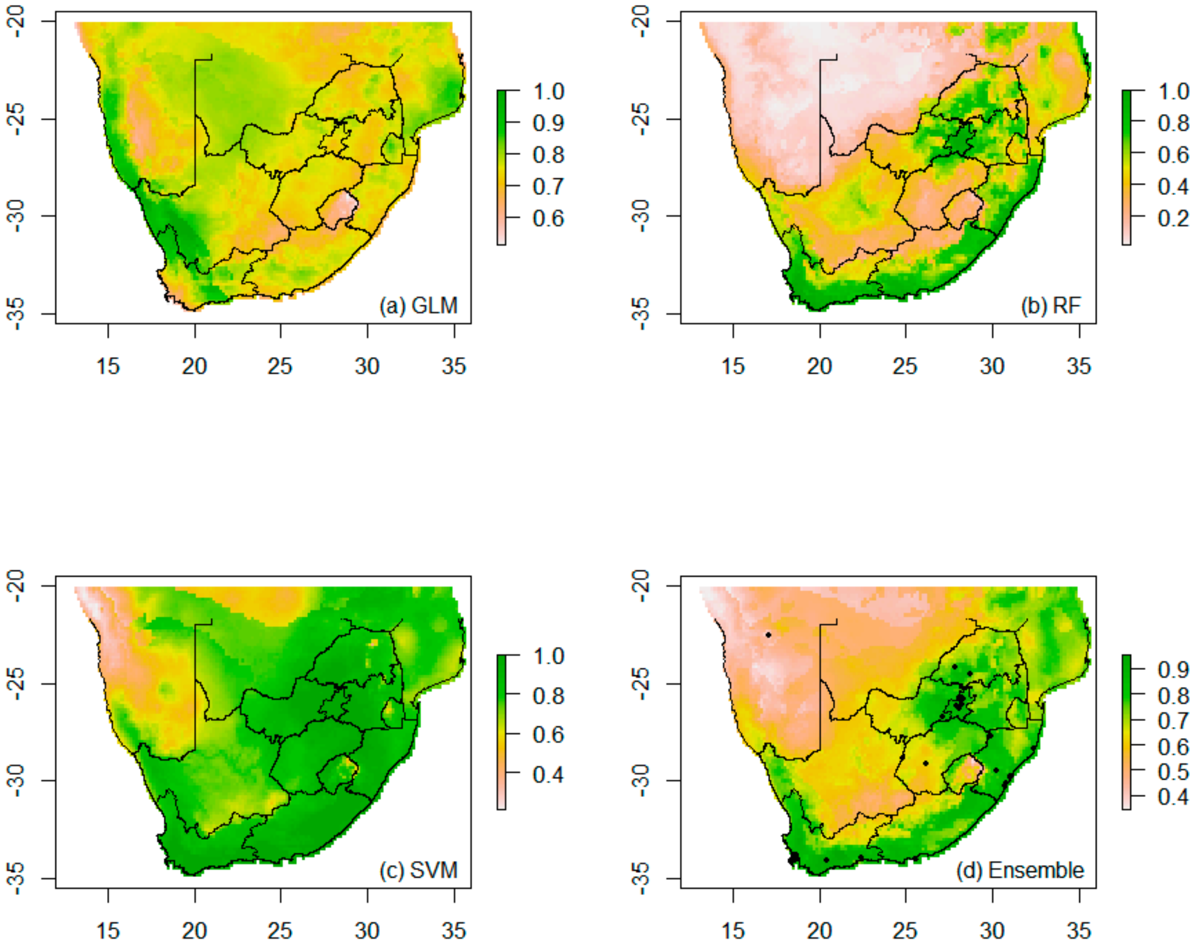

Figure S4. Ensemble species distribution modelling showing the potential distribution of the cockatiel (*Nymphicus hollandicus*) within South Africa. Black dots resemble the distribution localities of cockatiel reported lost in the country.

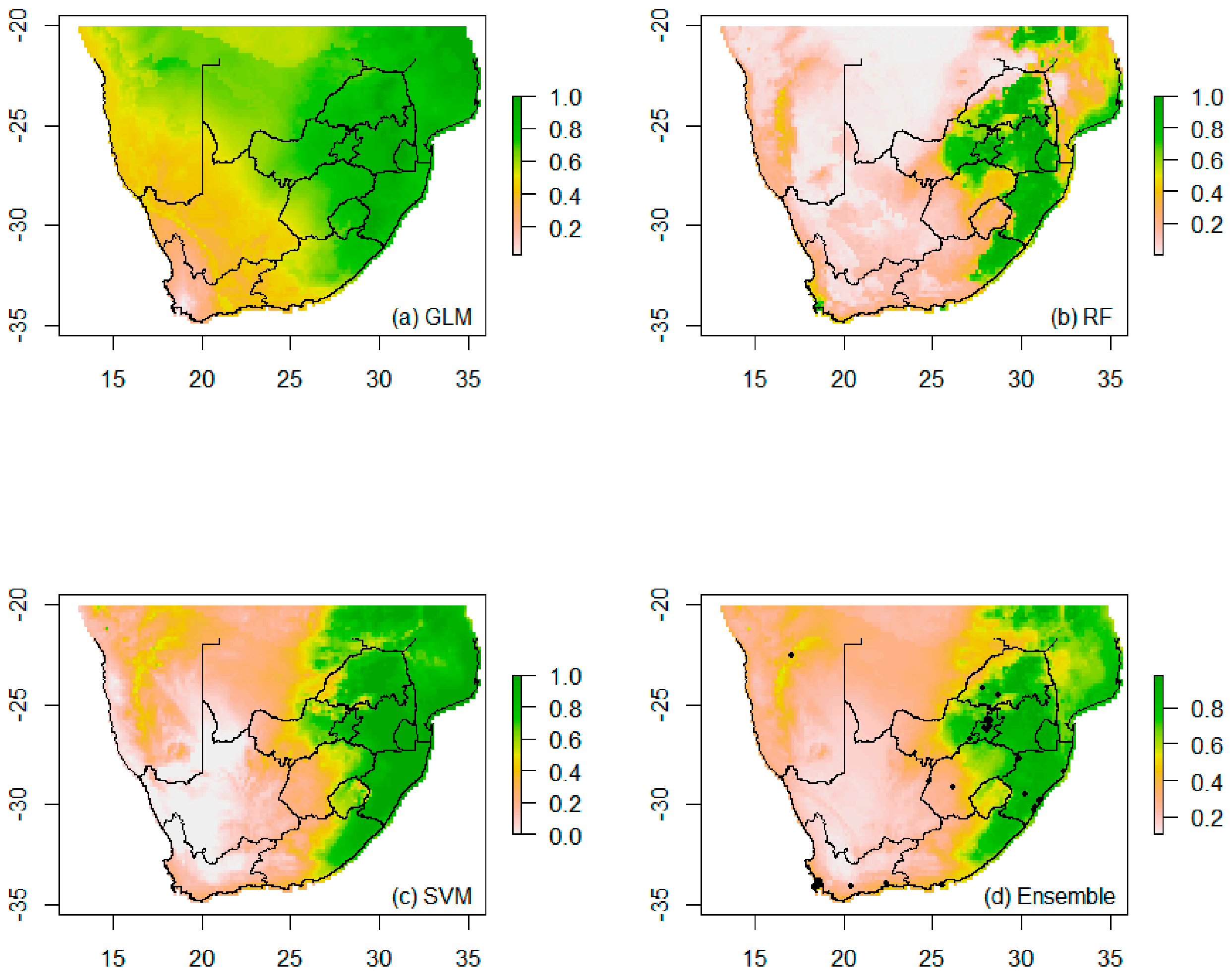

Figure S5. Ensemble species distribution modelling showing the potential distribution of the green-cheeked conure (*Pyrrhura molinae*) in South Africa. Black dots resemble the distribution localities of green-cheeked conure reported lost in the country.

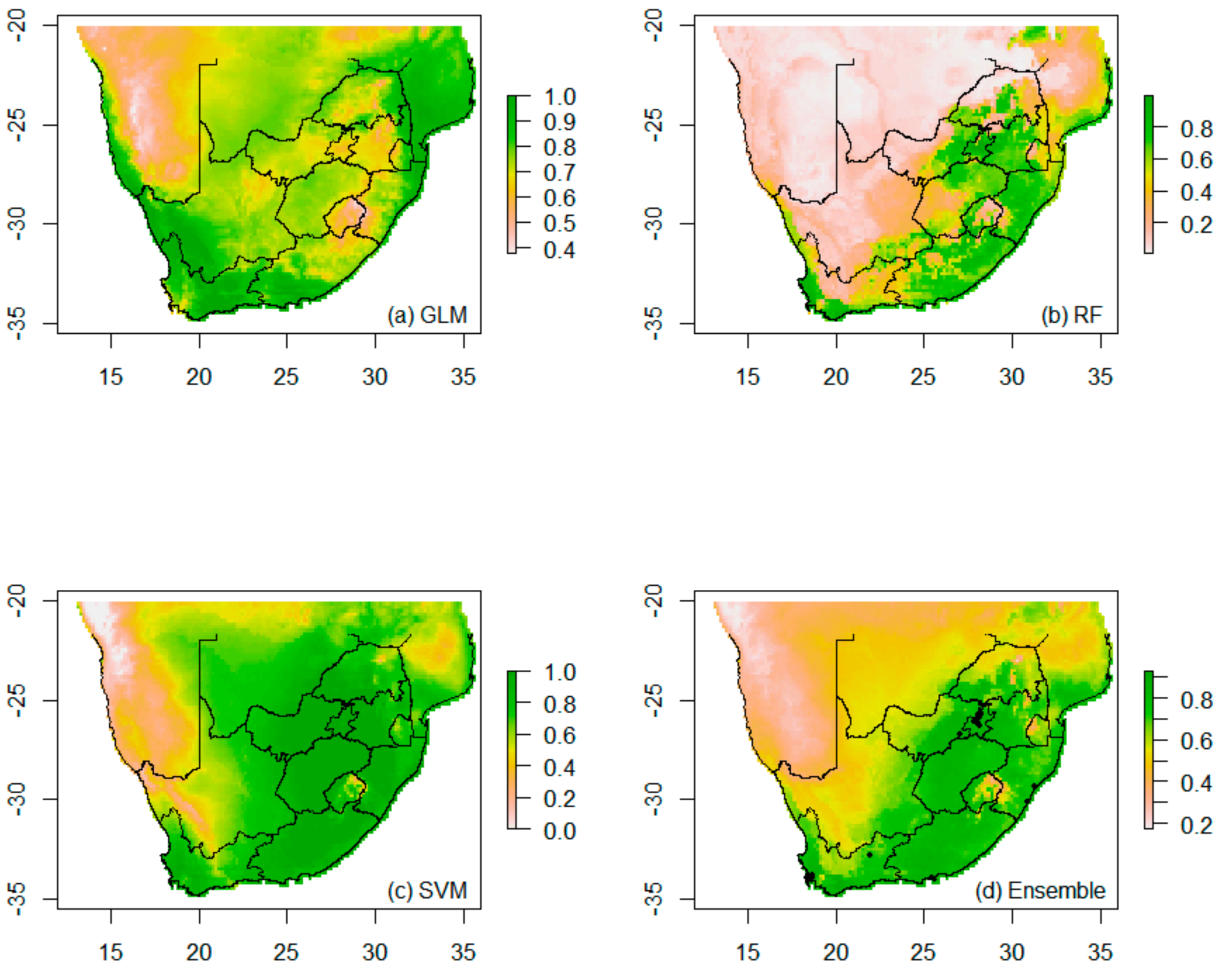

Figure S6. Ensemble species distribution modelling showing the potential distribution of the monk parakeet (*Myiopsitta monachus*) in South Africa. Black dots resemble the distribution localities of monk parakeets reported lost in the country.
